# Clinical efficacy and neural mechanisms of Tuina for primary dysmenorrhea with cold congestion and blood stasis pattern: a protocol for a randomized controlled fMRI trial

**DOI:** 10.3389/fmed.2026.1915832

**Published:** 2026-09-23

**Authors:** Yuchen Lin, Changzheng Jiang, Mimi You, Shuijin Chen, Hongye Huang, Huanzhen Zhang, Lechun Chen, Zhigang Lin

**Affiliations:** 1 Fujian University of Traditional Chinese Medicine, Fuzhou, Fujian, China; 2 Affiliated Rehabilitation Hospital of Fujian University of Traditional Chinese Medicine, Fuzhou, Fujian, China; 3 Fujian Key Laboratory of Rehabilitation Technology, Fuzhou, Fujian, China

**Keywords:** clinical efficacy, cold congestion and blood stasis pattern, controlled trial, neural mechanisms, PD, Tuina

## Abstract

**Background:**

Primary dysmenorrhea (PD) is one of the most common gynecological disorders in women of reproductive age and significantly impairs quality of life. Epidemiological data indicate that the cold congestion and blood stasis pattern accounts for a large proportion of PD cases. Tuina, a traditional Chinese external therapy, has unique advantages in pain management; however, its clinical efficacy and central mechanisms for PD remain unclear.

**Objective:**

This study aims to conduct an assessor-blind, parallel-group randomized controlled superiority trial, integrating functional magnetic resonance imaging (fMRI), to evaluate the clinical efficacy, safety, and central brain network modulation of Tuina therapy for primary dysmenorrhea with the cold congestion and blood stasis pattern.

**Methods:**

This study employed a randomized controlled trial design. A total of 54 patients who met the diagnostic criteria were recruited and randomly assigned in a 1:1 ratio to either the treatment or control group. An additional 15 healthy women were included as the healthy group. Participants in the treatment group received standardized manipulation over two consecutive menstrual cycles. For each cycle, treatment began 7 days before menstruation and was administered once daily for 7 consecutive days. Participants in the control group received conventional oral ibuprofen, taken as needed for pain relief. The primary outcome was the change in the Visual Analog Scale score. Secondary outcomes included the COX Menstrual Symptom Scale, Traditional Chinese Medicine pattern score, sleep index, and psychological assessments. All outcomes were evaluated at three time points: baseline, after the first treatment cycle, and after the second treatment cycle. All participants underwent two fMRI sessions—both resting-state and task-state fMRI scans—during days 1–3 of their menstrual cycle: once before enrollment and once after the treatment period. All statistical analyses followed the intention-to-treat principle.

**Discussion:**

This study aims to provide high-level clinical evidence on pain relief, symptom improvement, and brain functional network regulation for the treatment of cold congestion stasis type dysmenorrhea with Tuina therapy, using a randomized controlled trial design combined with functional magnetic resonance imaging.

**Clinical Trial Registration:**

https://itmctr.ccebtcm.org.cn, International Traditional Medicine Clinical Trial Registry ITMCTR2026001387.

## Introduction

Primary dysmenorrhea (PD) refers to recurrent lower abdominal or lumbosacral pain during menstruation in the absence of pelvic pathology. Accompanying symptoms include diarrhea, nausea, and fatigue, with severe cases potentially leading to syncope. The condition substantially impairs daily functioning, work productivity, and psychological wellbeing (1, 2). Pain typically peaks during maximum menstrual flow and persists for 2–3 days (3). Global prevalence ranges from 47% to 88%, with 2%–29% of individuals with PD reporting pain severe enough to disrupt daily activities (2, 4). Rising prevalence rates may be attributable to increasing life stress and emotional factors. Within the framework of Traditional Chinese Medicine (TCM), PD most commonly presents as the cold congestion and blood stasis pattern (5). This pattern arises from cold pathogens that induce stagnation and obstructed blood flow. Clinical manifestations include cold lower abdominal pain before or during menstruation that is relieved by warmth, dark menstrual blood with clots, chills, and cold extremities.

Non-steroidal anti-inflammatory drugs (NSAIDs) remain the first-line treatment for PD. While they provide rapid symptomatic relief, they do not address underlying etiologies, resulting in high recurrence rates. Their use is also associated with adverse effects, including gastrointestinal discomfort and reduced menstrual flow; severe cases may progress to amenorrhea (6). Traditional Chinese Medicine addresses these limitations through pattern differentiation, its core diagnostic principle. This holistic approach seeks to understand the nature of disease through comprehensive clinical analysis, shifting therapeutic focus from symptom suppression to targeting root causes with individualized treatment strategies.

The pathophysiology of PD involves complex interactions between neuroendocrine, inflammatory, and vascular responses. Estrogen–progesterone imbalance promotes prostaglandin and leukotriene synthesis, triggering excessive uterine contractions and vasospasm. This cascade leads to local ischemia, hypoxia, and pain signal generation (7, 8). Pain signal transmission can induce peripheral and central sensitization, further amplifying pain perception. Neuroimaging studies have confirmed structural and functional alterations in pain-related brain regions, including the anterior cingulate cortex and insula, in individuals with PD. These changes occur alongside disruptions in emotional regulation and cognitive processing networks. This may explain why nearly half of PD patients experience comorbid anxiety or depression, and why negative emotions show a significant positive correlation with pain intensity (9, 10).

Tuina therapy is a traditional Chinese manipulative technique grounded in the theories of yin–yang, qi and blood, zang–fu organs, and meridians. Its therapeutic principles involve harmonizing yin and yang, unblocking meridians, and strengthening the vital qi to expel pathogenic factors. Clinical evidence supports its efficacy and favorable safety profile (11). Within TCM, PD falls under the category of “menstrual abdominal pain.” The core pathogenesis involves cold pathogens impairing yang qi, leading to qi and blood stagnation. Cold congestion and blood stasis constitutes the predominant clinical pattern, with treatment focused on warming the meridians, dispersing cold, promoting blood circulation, and removing blood stasis (12). Modern research highlights the advantages of TCM in treating PD, including individualized pattern differentiation and multi-target intervention (13). Mechanistically, Tuina may alleviate smooth muscle spasm by regulating prostaglandin levels and improving uterine blood perfusion. In addition, it may modulate pain-related brain network functional connectivity (FC), thereby enhancing central pain processing and emotional regulation (14). This peripheral–central dual regulatory mechanism aligns with the holistic perspective of TCM and corroborates contemporary pathophysiological understanding, providing a theoretical basis for Tuina in PD management.

Despite promising clinical effects, evidence for Tuina in PD remains largely limited to observational studies. Its specific advantages over conventional treatments, particularly its central neuromodulatory mechanisms, have yet to be systematically established. This mechanistic gap limits the integration of Tuina into precision pain management paradigms. To address this gap, we designed a randomized controlled trial targeting PD patients with the cold congestion and blood stasis pattern, integrating fMRI with multidimensional clinical assessments. We hypothesize that Tuina not only effectively relieves pain and associated symptoms but also modulates functional connectivity within key brain regions, including the default mode network and limbic system, thereby restoring central balance between pain perception and emotional regulation. By establishing correlational models between clinical efficacy and neuroimaging biomarkers, this study aims to construct a comprehensive evidence chain that spans from peripheral symptom improvement to central functional remodeling. Findings may facilitate the transformation of TCM external therapy from empirical practice to mechanism-guided, precision-intervention paradigms.

## Materials and methods

### Trial strategy

This fMRI-integrated randomized controlled trial evaluated the clinical efficacy and central neural mechanisms of Tuina for PD with the cold congestion and blood stasis pattern. The protocol followed the Declaration of Helsinki and SPIRIT 2025 guidelines (15). Patients with this specific pattern were randomly assigned 1:1 to treatment or control groups. The treatment group received Tuina therapy 7 days before menstruation, once a day, for 7 consecutive days in each cycle, for a total of two cycles. The control group took medication orally as needed. When the Visual Analog Scale (VAS) score is ≥4, rescue analgesics may be used, and all medication usage must be recorded. All PD patients underwent resting-state fMRI before and after intervention. Healthy group patients received only one baseline scan. Baseline data were collected for covariate analysis, including menstrual characteristics, VAS scores, prior analgesic use, and treatment history. Table 1 presents the schedule for enrollment, interventions, and assessments. Figure 1 illustrates the study design flowchart. Figure 2 shows photographs of participants during the MRI scanning procedure.

**Table 1:** Schedule of enrollment, interventions, and assessment.

Visit item	Enrollment	Allocation	Treatment visit		Follow-up period
Timepoint	Menstrual cycle 0	Menstrual cycle 0	Menstrual cycle 1	Menstrual cycle 2	Menstrual cycle 3
Enrollment					
Signed the informed consent form	√				
General information		√			
Inclusion and exclusion criteria		√			
Randomization		√			
Demographics, medical history, history of present illness		√			
Interventions					
Treatment group (*n* = 27)			√	√	
Control group (*n* = 27)			√	√	
Healthy group (*n* = 15)			×	×	
Efficacy observation					
VAS		√	√	√	√
CMSS		√	√	√	√
TCM pattern score		√	√	√	√
SAS		√	√	√	√
SDS		√	√	√	√
PSQI		√	√	√	√
fMRI		√		√	
Safety evaluation					
Adverse event			Fx1 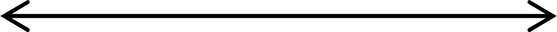		

VAS, Visual Analog Scale; CMSS, Cox Menstrual Symptom Scale; TCM pattern score, Traditional Chinese Medicine pattern score; SDS, Self-Rating Depression Scale; PSQI, Pittsburgh Sleep Quality Index; fMRI, functional magnetic resonance imaging.

**Figure 1:**
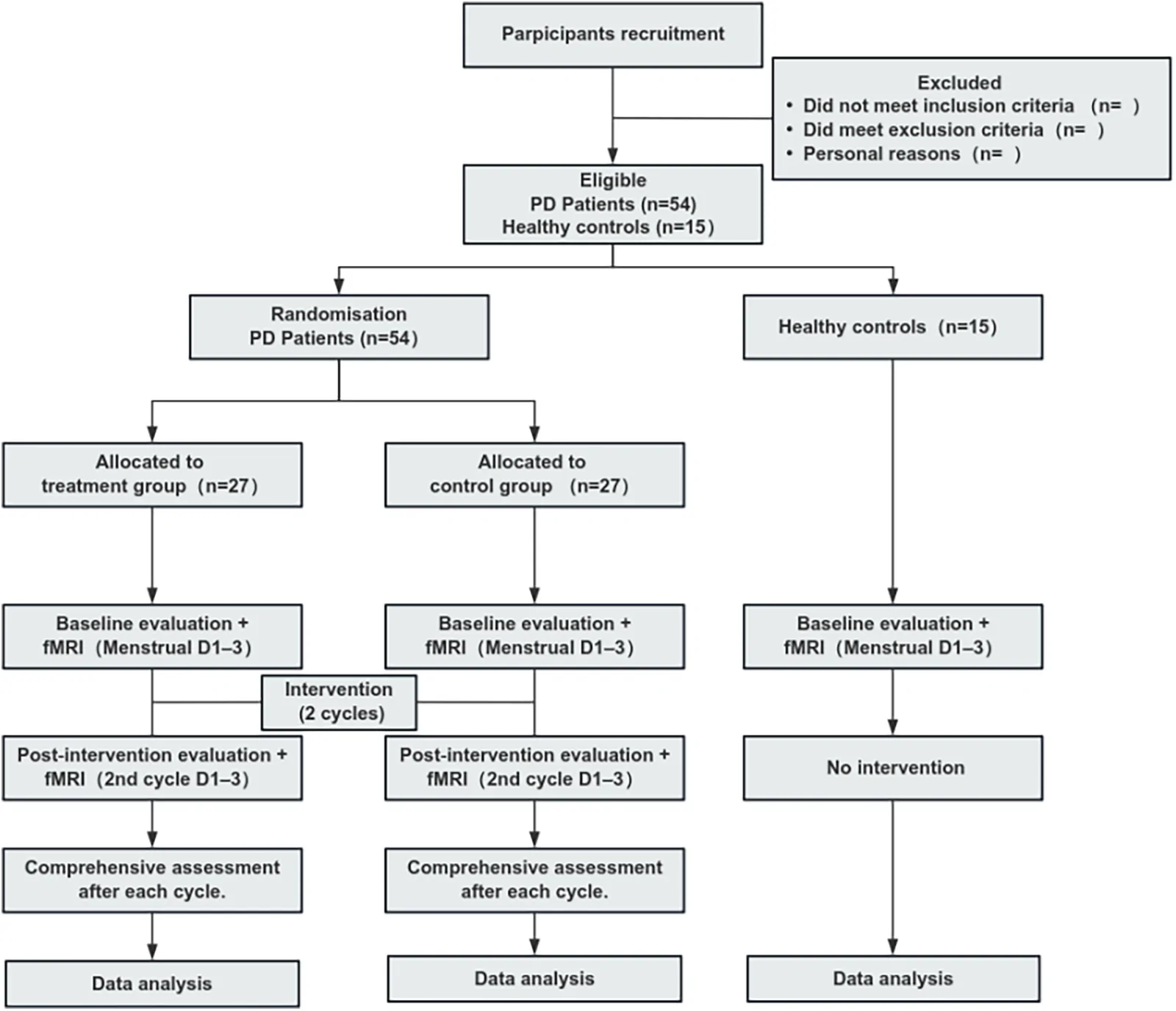
Study flowchart. PD, primary dysmenorrhea; VAS, Visual Analog Scale; fMRI, functional magnetic resonance imaging; ITT, intention-to-treat.

**Figure 2:**
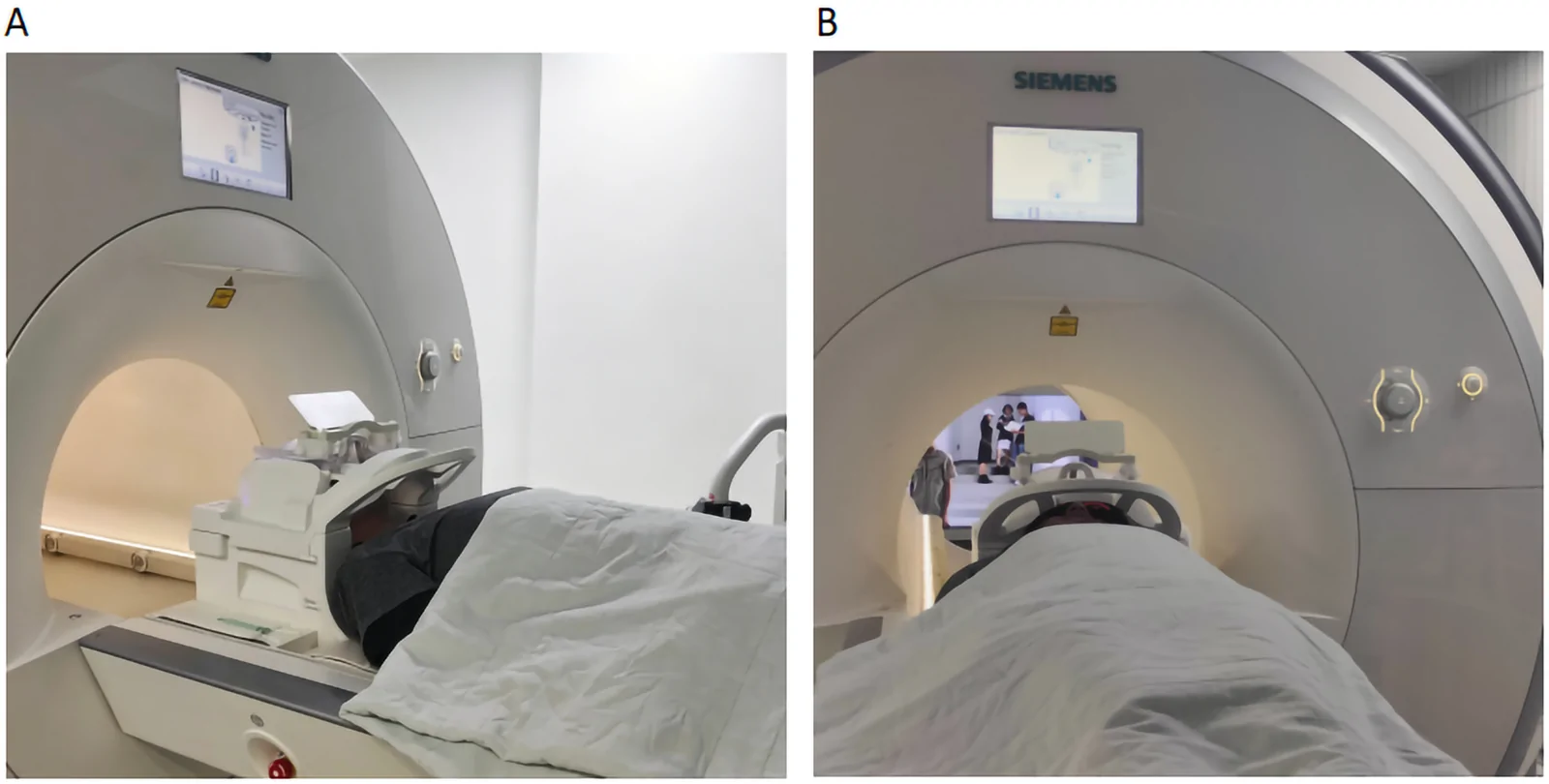
MRI scanning procedure. **(A)** Participant positioning and head stabilization during MRI scanning; **(B)** task-state fMRI acquisition.

### Recruitment and informed consent

This study was conducted at the outpatient clinic of the Affiliated Rehabilitation Hospital of Fujian University of Traditional Chinese Medicine. We planned to recruit 54 PD patients who met the cold congestion and blood stasis pattern criteria, alongside 15 healthy female controls. Recruitment was implemented via hospital social media and outpatient notices. Prior to enrollment, all eligible participants provided written informed consent; researchers fully outlined study procedures, interventions, risks, and benefits, with specific emphasis on fMRI safety and Tuina protocols. Written guardian consent is mandatory for adolescent participants under 18 years of age. Participants may withdraw unconditionally at any stage without compromising medical rights. Baseline data were collected for all participants, including demographic and clinical characteristics, such as age, height, weight, body mass index (BMI), education level, occupation, disease duration, medical history (with focus on gynecological and systemic disorders), and menstrual cycle parameters (cycle length, regularity, and menstrual duration). Vital signs (blood pressure, pulse, and body temperature) were also documented. All data were obtained via standardized questionnaires and physical examinations and used for covariate adjustment and baseline comparability analysis between groups. All data were strictly anonymized, stored in a password-protected database, and used exclusively for this study. The protocol was approved by the Ethics Committee of the Affiliated Rehabilitation Hospital of Fujian University of Traditional Chinese Medicine (Approval No. 2025KY-033-01).

### Eligibility criteria

To be eligible, individuals must meet all inclusion criteria and none of the exclusion criteria during screening.

### Inclusion criteria

PD patients: (1) nulliparous women aged 15–30 years with PD; (2) right-handed; (3) meeting conventional PD diagnostic criteria; (4) meeting TCM criteria for the cold congestion and blood stasis pattern; (5) regular menstrual cycle (28 ± 7 days); (6) no pelvic pathology on examination or imaging; (7) menstrual pain for ≥3 cycles, with a VAS score of ≥4; (8) no analgesics within 24 h before fMRI; no analgesics, sedatives, or hormones within 1 week prior; and no oral contraceptives within 3 months prior; and (9) voluntary participation with written informed consent.

Healthy participants: (1) nulliparous women aged 15–30 years; (2) right-handed; (3) regular menstrual cycle (28 ± 7 days); (4) no pelvic pathology on examination or imaging; (5) no analgesics within 24 h before fMRI; no analgesics, sedatives, or hormones within 1 week prior; no contraceptives within 3 months prior; (6) no significant menstrual pain, with a VAS score of 0; and (7) voluntary participation with written informed consent.

### Exclusion criteria

Exclusion criteria included (1) secondary dysmenorrhea or dysmenorrhea related to intrauterine device use; (2) pregnancy or planned pregnancy; (3) a history of cardiovascular, cerebrovascular, or psychiatric disorders; (4) contraindications to fMRI; (5) allergy or adverse reactions to ibuprofen sustained-release capsules; and (6) alcohol or drug dependence.

### Withdrawal criteria

Withdrawal criteria included (1) intercurrent symptoms, allergies, or adverse events (AEs) requiring treatment discontinuation; (2) concurrent use of other medications or treatments during trial that may affect outcomes; (3) poor compliance with scheduled treatments; and (4) voluntary withdrawal or loss to follow-up.

### Randomization and blinding

Randomization was performed by an independent statistician not involved in intervention delivery. A random sequence was generated in SPSS 27.0, and 54 eligible patients were allocated 1:1 to either the treatment or control group. The 15 healthy controls did not undergo randomization or blinding, as they received no intervention and served only as a baseline reference. The allocation was sealed in an opaque envelope and stored by researchers independent of recruitment and outcome assessment. Envelopes were opened sequentially at enrollment to assign group allocation. Given the nature of manual Tuina therapy, blinding of practitioners and participants was not feasible. To ensure evaluative objectivity, an assessor-blind design was implemented. All outcome assessments, including questionnaire administration, fMRI scanning, and imaging processing, were conducted by researchers blinded to group assignment. Statistical analysts also remained blinded until the completion of data analysis.

### Interventions

The intervention was conducted in accordance with CONSORT guidelines (16). The control group received ibuprofen sustained-release capsules as recommended by international clinical practice guidelines for PD (4). The Tuina protocol was developed based on prior clinical trials and established clinical experience (17–20), with acupoint selection and manipulative techniques referenced from the national textbook *Tuina Therapeutics*. All acupoints were located in strict accordance with the WHO Standard Acupuncture Point Locations (43). Acupoint locations are illustrated in Figure 3 and Table 2.

**Figure 3:**
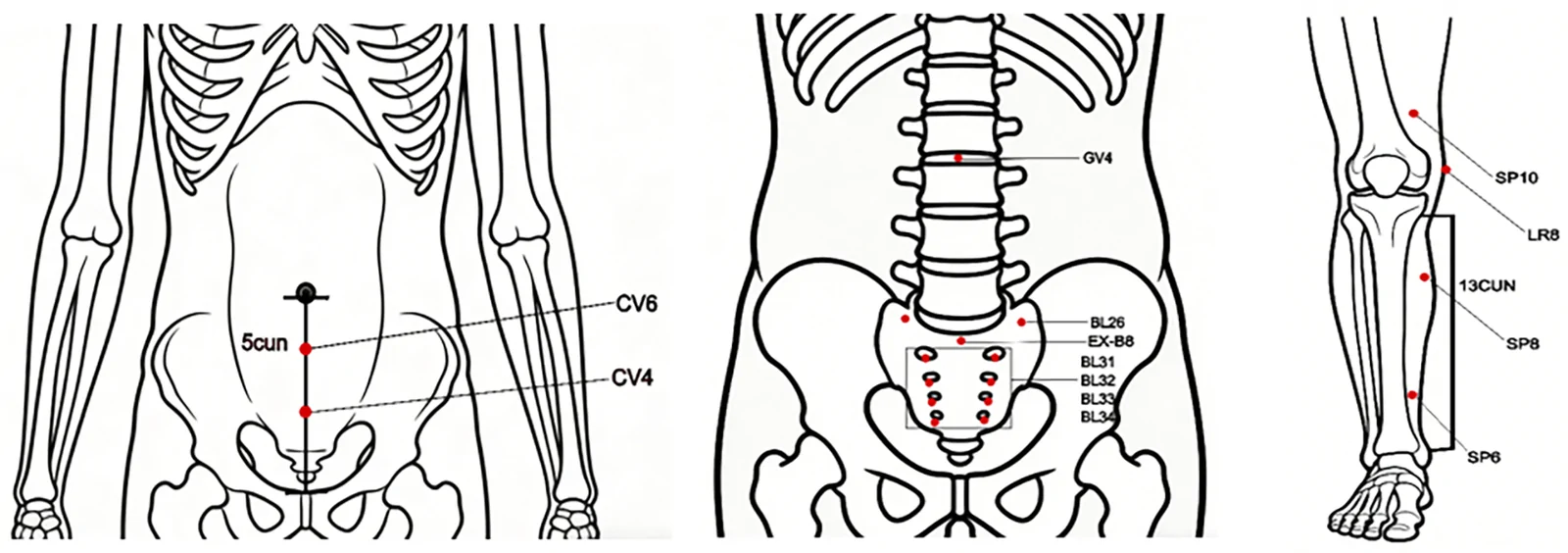
Location of acupoints.

**Table 2:** Location of acupoints.

Acupoints	Location
CV4 (Guanyuan)	On the anterior midline of the lower abdomen, 3 B-cun inferior to the umbilicus
CV6 (Qihai)	On the anterior midline of the lower abdomen, 1.5 B-cun inferior to the umbilicus
GV4 (Mingmen)	On the posterior midline, in the depression below the spinous process of the 2nd lumbar vertebra (L2)
EX-B8 (Shiqizhui)	On the posterior midline, in the depression below the spinous process of the 5th lumbar vertebra (L5)
BL26 (Guanyuanshu)	On the back, in the depression below the spinous process of the 5th lumbar vertebra (L5), 1.5 B-cun lateral to the posterior midline
BL31 (Shangliao)	On the sacrum, in the first posterior sacral foramen, approximately 0.5–1 B-cun lateral to the posterior midline
BL32 (Ciliao)	On the sacrum, in the second posterior sacral foramen, approximately 0.5–1 B-cun lateral to the posterior midline
BL33 (Zhongliao)	On the sacrum, in the third posterior sacral foramen, approximately 0.5–1 B-cun lateral to the posterior midline
BL34 (Xialiao)	On the sacrum, in the fourth posterior sacral foramen, approximately 0.5–1 B-cun lateral to the posterior midline.
SP10 (Xuehai)	On the medial aspect of the thigh, 2 B-cun superior to the medial epicondyle of the femur, at the prominence of the vastus medialis muscle
SP6 (Sanyinjiao)	On the tibial aspect of the leg, posterior to the medial border of the tibia, 3 B-cun superior to the prominence of the medial malleolus
SP8 (Diji)	On the tibial aspect of the leg, posterior to the medial border of the tibia, 3 B-cun inferior to SP9 (Yinlingquan)
LR8 (Ququan)	On the medial aspect of the knee, when the knee is flexed, at the medial end of the popliteal crease, posterior to the medial epicondyle of the femur, in the depression anterior to the tendons of the semitendinosus and semimembranosus muscles

### Treatment group

Tuina was performed by a licensed TCM physician, with over 8 years of clinical experience in Tuina practice. The intervention targeted the lumbosacral, abdominal, and lower limb regions. Selected acupoints included Guanyuanshu (BL26), Shiqizhui (EX-B8), Baliao (BL31-34), Mingmen (GV4), Guanyuan (CV4), Qihai (CV6), Xuehai (SP10), Sanyinjiao (SP6), Diji (SP8), and Ququan (LR8). The standardized procedure is as follows: participants lie in the prone position while the therapist applies pressing and kneading to BL26 and EX-B8 for 1 min each, followed by rolling manipulation along the lumbosacral region and bilateral paraspinal areas for 2 min. Palm-rubbing is then performed on the lumbosacral area and BL31–34 until local warmth is achieved, followed by hypothenar rubbing applied to GV4 to induce a deep warming sensation. Participants then assume the supine position. The therapist performs clockwise circular rubbing on the abdomen for 5 min, followed by palm-kneading until warmth and comfort are attained. CV4 and CV6 are then pressed and kneaded for 1 min each. Finally, pressing and kneading are applied to the lower limb acupoints SP10, SP6, SP8, and LR8 for 1 min each. Treatment is initiated 7 days before menstruation, administered once daily for 30 min per session. One course consists of seven consecutive sessions covering one menstrual cycle, and a total of two courses are administered.

### Control group

The control group received standardized medication: ibuprofen sustained-release capsules (0.3 g/capsule; manufacturer: Nanjing Yiheng Pharmaceutical Co., Ltd., Nanjing, Jiangsu Province, China; batch number: 2503B015), administered orally on an as-needed basis. The dose is 0.3 g per administration, taken twice daily at a 12-h interval. Medication is initiated at the onset of dysmenorrhea and discontinued once pain relief is achieved. Participants are required to record their medication use over two consecutive menstrual cycles using a medication log (Table 3), documenting the date, time, dose, and changes in pain intensity for each administration, to assess medication adherence.

**Table 3:** Medication log.

No.	Date (YYYY/MM/DD)	Time of medication (24 h）	Dose (g)	Frequency (per day)	Dosing interval (h)	Time to pain relief	VAS before dose	VAS 1 h after dose
1	_____/_____/____ (year/month/day）	______ ：______						
2	_____/_____/____ (year/month/day	______ ：______						
3	_____/_____/____ (year/month/day	______ ：______						

### Healthy group

The healthy control group received no intervention. They underwent a single MRI scan during days 1–3 of menstruation at enrollment and complete the associated symptom assessment scales.

### Emergency treatment

All AEs are closely monitored throughout the study. Potential risks include local bruising, increased pain, or a needling-like sensation from Tuina; gastrointestinal discomfort, nausea, headache, or drowsiness from ibuprofen; and claustrophobia, dizziness, or fatigue associated with fMRI scanning. If any adverse reactions occurred, symptomatic treatment was provided, and the study physician assessed whether to reduce the dose, temporarily discontinue the medication, or permanently withdraw it. All AEs were managed immediately and documented in the Case Report Forms (CRFs), including onset time, management, and outcome. For participants in the treatment group, if the VAS score was ≥4 during treatment, participants could take ibuprofen sustained-release capsules as needed, and this was recorded in the medication log. If pain persisted or became intolerable, the intervention was discontinued and medical support was provided. For control group participants with no pain relief after standard medication, comprehensive interventions might be offered following evaluation. All AE and emergency records were submitted to the Ethics Committee. Serious AEs were reported within 24 h. The causality and severity of each AE were continuously assessed to ensure participant safety and data integrity. All emergency management and rescue medication details were documented in the CRFs by researchers and included in the final analysis.

### Primary outcome

The VAS (21) score served as the primary outcome measure for pain intensity. It comprises a 10-cm horizontal line, with “0” (no pain) and “10” (worst possible pain) anchored at each end. Participants marked the point corresponding to their current pain intensity, with higher scores indicating more severe pain. The VAS assessed at four predefined time points: baseline, after the first and second menstrual cycles of intervention, and at the 1-month follow-up.

### Secondary outcomes

#### Associated symptom assessment

The Cox Menstrual Symptom Scale (CMSS) (22) includes 18 common menstrual symptoms, such as lower abdominal pain, nausea, vomiting, lumbosacral soreness, fatigue, and dizziness. Each symptom was assessed on two dimensions: duration and severity. Duration is scored from 0 to 4 (0 = none, 1 ≤ 3 h, 2 = 3–7 h, 3 = 7–24 h, 4 ≥ 24 h). Severity is also scored from 0 to 4 based on the degree of disruption to daily activities.

The TCM Pattern Score (23) is a validated instrument used to evaluate the characteristics and severity of the cold congestion and blood stasis pattern in patients with dysmenorrhea. Developed according to the Guiding Principles for Clinical Research of New Chinese Medicines and traditional Chinese medicine diagnostic criteria, this scale quantifies core symptoms and signs, including cold lower abdominal pain during menstruation relieved by warmth, scanty menstrual flow, dark purple menstrual blood with clots, chills, cold extremities, pale complexion, nausea, and vomiting. Each symptom is rated on a four-point scale: none, mild, moderate, and severe, with corresponding scores of 0, 2, 4, and 6, respectively. The total score is calculated as the sum of all item scores; higher scores indicate more severe pattern severity. This score is used to evaluate changes in pattern status before and after intervention, with a reduction in score indicative of therapeutic effect. All TCM pattern assessments were performed by the same trained TCM physician to ensure diagnostic consistency.

The Self-Rating Anxiety Scale (SAS) (24–26) is a self-report psychological assessment tool used to evaluate the severity of an individual’s anxiety symptoms and their changes over the past week. This self-rated scale includes 20 items covering tension, fear, and autonomic symptoms. Higher scores indicate more severe anxiety.

The Self-Rating Depression Scale (SDS) (25–27) is a self-report psychological assessment tool used to evaluate the severity of a person's depressive symptoms and how they have changed over the past week. It includes 20 self-rated items covering depressed mood, somatic symptoms, psychomotor retardation, and cognitive impairment. It can sensitively reflect the severity and dynamic changes of depression.

The Pittsburgh Sleep Quality Index (PSQI) (28) measures subjective sleep quality over the past month. It includes 19 self-rated items grouped into 7 components: subjective sleep quality, sleep latency, sleep duration, habitual sleep efficiency, sleep disturbances, use of sleeping medication, and daytime dysfunction. A PSQI score >5 indicates poor sleep quality; higher scores reflect worse sleep.

The above scales were assessed at four predefined time points: baseline, after the first and second menstrual cycles of intervention, and at the 1-month follow-up.

#### Brain fMRI

We employed a multimodal fMRI protocol combining resting-state and task-state scans on a Siemens 3.0 T MAGNETOM Prisma scanner (Germany) with a 64-channel head-neck coil. PD patients underwent two scans: one at baseline (days 1–3 of the first menstrual cycle after enrollment) and one post-treatment (days 1–3 of the first menstrual cycle following two intervention cycles). Healthy controls received a single baseline scan during days 1–3 of their menstrual cycle. Before each scan, participants removed all metal objects. Foam pads minimized head motion, and earplugs reduced noise. Participants stayed awake with eyes closed, relaxed, and avoided systematic thinking; they could stop at any time if uncomfortable.

The MRI scanning protocol includes three sequences: first, high-resolution 3D T1-weighted structural imaging for brain localization and spatial normalization (repetition time [TR] = 2,200 ms, echo time [TE] = 2.48 ms, flip angle [FA] = 8°, field of view [FOV] = 230 × 230 mm², matrix = 256 × 256, voxel size = 0.98 × 0.98 × 1.0 mm³, 160 slices); second, resting-state blood oxygen level-dependent (BOLD) -fMRI for functional network analysis (TR = 2000ms, TE = 30 ms, FA = 90°, FOV = 230 × 230 mm^2^, matrix = 64 × 64, voxel size = 3.6 × 3.6 × 3.6 mm^3^, 37 slices, 10 min, 300 time points); and third, task-state fMRI with two modules assessing physical pain and social pain empathy. Participants rated visual stimuli on a 1–4 scale (each rating ∼5 s), and VAS scores were recorded immediately before and after each module to link brain activation with subjective pain experience. Each task-state module lasted approximately 10 min, with a brief rest interval between modules, totaling approximately 20 min for the two task-state modules. The total scanning session, including localizer, structural imaging, resting-state fMRI (10 min), and task-state modules, took approximately 35–40 min per participant. To minimize fatigue and motion, foam pads were used for head stabilization, and participants were monitored via intercom throughout the scan; they could rest or suspend the scan at any time by squeezing a panic ball if they feel fatigued or uncomfortable.

#### Data management and quality control

All clinical data were recorded on standardized paper CRFs and promptly entered into electronic databases for secure online storage to ensure accuracy, completeness, and timeliness. Neuroimaging data were backed up to an encrypted dedicated hard drive immediately after each scan and stored under de-identified codes. The Ethics Committee of the Affiliated Rehabilitation Hospital of Fujian University of Traditional Chinese Medicine oversaw the entire study and retained the final authority to suspend or terminate the trial based on safety and ethical concerns.

### Sample size

Sample size estimation was based on the VAS score as the primary outcome. A reduction in VAS score of 1–2 points was deemed clinically meaningful (29). Previous Tuina trials for PD have reported effect sizes of 0.8–1.0 (30); we therefore adopted a moderate-to-large effect size (d = 0.8). Using G*Power 3.1 software with a two-independent-samples *t*-test model (two-sided *α* = 0.05, power = 0.80), the required sample size was calculated to be 21 participants per group. To account for an anticipated 20% dropout rate, we calculated 27 participants per group (21/0.8 = 26.25, rounded up to 27), yielding a total of 54 patients. This final sample size also met the general recommendation of 12–15 participants per group for fMRI studies (31, 32) and further accounted for potential data loss due to excessive head motion or other imaging artifacts. In addition, 15 healthy women were recruited as a neuroimaging reference control group. Patients were randomly allocated 1:1 to the treatment or control group (*n* = 27 per group). An additional 15 healthy women served as a neuroimaging reference control.

### Clinical data analysis

All statistical analyses were performed using SPSS 27.0. Categorical data are presented as percentages or proportions. Normally distributed continuous data are expressed as mean ± standard deviation (SD), while non-normally distributed data are presented as median and interquartile range (IQR). The study strictly adhered to the intention-to-treat (ITT) principle, including all randomized participants who provided at least one baseline outcome measure, regardless of subsequent treatment adherence or withdrawal. For primary and secondary outcomes measured repeatedly over multiple time points, a linear mixed-effects model was used for longitudinal analysis. The model included group, time point, and their interaction as fixed effects, with the baseline value of the corresponding outcome served as a covariate. Between-subject variability was accounted for by specifying an appropriate random-effects structure. This model effectively utilizes incomplete data under the missing-at-random assumption, providing robust estimates of treatment effects without relying on a single imputation strategy. To further assess the robustness of the results to missing-data assumptions, multiple imputation by chained equations (MICE) was performed, assuming missingness is at random (MAR). Multiple complete datasets were generated, the primary analysis model was refitted for each, and the point estimates and standard errors were combined according to Rubin's rules. Effect sizes and their 95% confidence intervals (CIs) were calculated to aid clinical interpretation. Between-group baseline characteristics and other secondary variables were compared using independent *t*-tests or Wilcoxon rank-sum tests, and chi-square or Fisher's exact tests, as appropriate. Incidence of adverse events was compared between groups using chi-square or Fisher's exact tests. When multiple comparisons were performed, the significance level was adjusted using the Bonferroni correction. All statistical tests were two-sided, and a *P*-value <0.05 was statistically significant.

### fMRI data analysis

Neuroimaging data preprocessing and analysis were performed by an independent statistician using SPM12 (Statistical Parametric Mapping, version 12), along with the CONN toolbox in MATLAB 2022b. Standard preprocessing steps were performed in sequence, including slice-timing correction, head motion correction, spatial normalization to the MNI152 standard space, spatial smoothing with a 6-mm full-width at half-maximum Gaussian kernel, and detrending. After preprocessing, functional changes in the brain were analyzed at three levels: first, local brain activity measured by the amplitude of low-frequency fluctuations (ALFF) and regional homogeneity (ReHo); second, FC using seed-based whole-brain FC analysis, where seeds were regions with significant group differences preliminarily identified at baseline. Statistical inference used a hierarchical thresholding strategy: the voxel-level threshold was set at *P* <0.005 uncorrected, followed by cluster-level false discovery rate correction, with a cluster-level *P* <0.05 considered statistically significant.

Two-sample *t*-tests were used for between-group comparisons, paired *t*-tests were used for within-group pretreatment versus post-treatment comparisons, and analysis of variance was used for multiple-group comparisons when applicable. To further explore associations between brain functional changes and clinical improvement, ALFF, ReHo, and FC values from brain regions with significant alterations were extracted. These values were correlated with the pre- to post-treatment changes in VAS scores and TCM pattern scores using Pearson's correlation analysis, or Spearman's rank correlation analysis if the data did not meet the normality assumption, thereby elucidating the central neuromodulatory mechanisms of Tuina therapy.

## Discussion

PD with the cold congestion and blood stasis pattern is a common condition affecting women of reproductive age. Tuina, a safe non-pharmacological external therapy in Traditional Chinese Medicine, relieves pain by warming and unblocking meridians, dispersing cold, and resolving stasis, with proven clinical efficacy (33). However, dysmenorrhea involves peripheral and central sensitization closely linked to brain functional remodeling. The central mechanisms of Tuina for PD, particularly its specific effects on brain functional networks, remain poorly understood, and high-level evidence remains lacking. fMRI offers a non-invasive tool to visualize brain responses to Tuina intervention.

The pathogenesis of PD begins with the excessive release of inflammatory mediators, such as prostaglandin F2α (PGF2α), in the endometrium during menstruation. This release triggers uterine smooth muscle spasms and vasoconstriction, leading to endometrial ischemia and the generation of pain signals (34). These nociceptive signals are transmitted to the spinal cord via peripheral afferent nerves, and long-term stimulation can induce central sensitization, further amplifying pain perception. Neuroimaging studies have further revealed neuroplastic changes in PD patients. Brain regions involved in pain processing and emotion regulation, including the anterior cingulate cortex, insula, and amygdala, show altered gray matter volume and impaired functional connectivity. In addition, the default mode network and salience network exhibit abnormal activity, accompanied by the reduced function of the descending pain-inhibitory system (35). Collectively, peripheral inflammation, spinal sensitization, and central dysregulation form a vicious cycle of pain–emotion interaction. This explains the neural basis for the high comorbidity of pain with anxiety and depression in PD patients, which is consistent with their clinical characteristics.

Current evidence suggests that Tuina may relieve primary dysmenorrhea through multiple pathways. Biochemically, it may regulate prostaglandin levels by reducing PGF2α and restoring the PGF2α/PGE2 balance, thereby directly alleviating excessive uterine contractions (36); it may also improve uterine and pelvic blood flow, reduce vascular resistance, and increase local oxygen supply, potentially breaking the ischemia–spasm–pain cycle (37). Structurally, techniques that release myofascial trigger points in the abdomen and lumbosacral region, along with spinal and pelvic adjustments, may effectively relieve muscle tension and spasm, correct unstable joint misalignments, and improve the local biomechanical environment (38, 39). Neurally, the abundant benign somatosensory inputs generated by Tuina may inhibit pain signal transmission via the spinal gate control mechanism and promote the release of endogenous analgesics, thereby exerting modulatory effects at the central level (40).

fMRI, based on BOLD signals, non-invasively maps brain activity with high spatial resolution by detecting local changes in blood oxygenation and flow induced by neuronal firing. This technique offers an objective, visual means of exploring the central effects of Tuina (41). In this study, we combined resting-state and task-state fMRI to dissect the underlying brain mechanisms. Resting-state fMRI captures spontaneous brain activity in the absence of a specific task, revealing the organization and connectivity of intrinsic functional networks. It is particularly suited for assessing baseline abnormalities in brain networks associated with chronic pain and for tracking network reorganization after intervention (42). Task-state fMRI, in contrast, evokes responses in relevant brain regions by having participants perform specific tasks, thereby directly mapping dynamic brain activity associated with cognitive and emotional processing (9). Together, the two approaches allow us to clarify, from a “static” network perspective, how Tuina reshapes the overall functional architecture of the brain while also revealing, from a “dynamic” response perspective, how it modulates pain-related cognitive and emotional processes.

To clarify the clinical efficacy of Tuina for primary dysmenorrhea with the cold congestion and blood stasis pattern and its effects on central pain networks, we designed a randomized controlled trial incorporating fMRI to systematically evaluate clinical outcomes and explore the relationship between functional brain changes and symptom relief. To ensure reliability and reproducibility, we implemented rigorous quality control at key stages. During recruitment, we applied strict inclusion and exclusion criteria based on international guidelines and TCM pattern diagnosis; in addition to demographic data, we systematically recorded menstrual cycle characteristics, recent medication use, psychological status, and sleep quality to control for potential confounders. During intervention, uniformly trained Tuina practitioners performed standardized manipulations to ensure consistency. For data acquisition, all MRI scans were scheduled within a fixed time window of the menstrual cycle, with standardized prescan preparation and in scan instructions; participants were asked to refrain from analgesics for 24 h before scanning to minimize interference from drug metabolism, physiological fluctuations, and head motion artifacts in neuroimaging data. Through these multi-level quality control measures, we aimed to objectively and reliably elucidate the peripheral and central integrative mechanisms of Tuina for dysmenorrhea, thereby providing high-quality evidence for the clinical application of this traditional Chinese external therapy.

Tuina has shown clear advantages in relieving clinical symptoms of dysmenorrhea, but its central neural mechanisms remain poorly understood, and related neuroimaging studies are still exploratory. Grounded in the TCM theory of cold congestion and blood stasis, this study systematically combined Tuina intervention with multimodal fMRI (resting-state and task-state), focusing on the most common clinical pattern of primary dysmenorrhea. We aimed to elucidate the central effects of Tuina on dysmenorrhea from a neuroscience perspective, providing new evidence for the modern mechanistic study of this traditional external therapy.

At the same time, this study has several limitations. Due to time and budget constraints, the intervention lasted only two menstrual cycles, and the sample size was limited, which may affect a full assessment of medium-to-long-term efficacy and stability. Because of the nature of Tuina manipulation, blinding of practitioners and participants was not feasible, introducing a risk of expectation bias. The follow-up period was relatively short, so the long-term persistence of Tuina's central neuromodulatory effects could not be fully examined. It should also be noted that the healthy control group only provides cross-sectional baseline data for comparison with baseline data of PD patients to confirm that the brain functional networks of the patient group are indeed abnormal, rather than for pre–post change comparisons.
